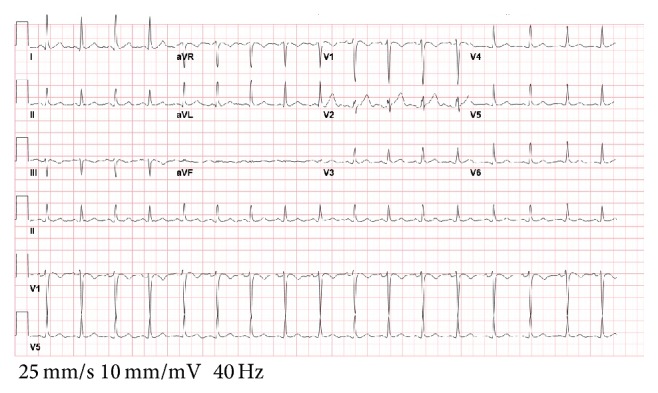# Small Bowel Obstruction Mimicking Acute ST-Elevation Myocardial Infarction

**DOI:** 10.1155/2015/739147

**Published:** 2015-03-08

**Authors:** Kunal Patel, Nai-Lun Chang, Oleg Shulik, Joseph DePasquale, Fayez Shamoon

**Affiliations:** Seton Hall University School of Health and Medical Sciences, Saint Michael's Medical Center, 111 Central Avenue, Newark, NJ 07109, USA

## Abstract

We present a case of a 42-year-old female who presented to our institution with a small bowel obstruction and had emergent surgical decompression. Thirteen days postoperatively, the patient became tachycardic and had worsening epigastric pain. Electrocardiogram showed significant ST-segment elevations in leads II, III, aVF, and V3–V6, suggesting the possibility of acute inferolateral myocardial infarction. Subsequent workup revealed the cause of the ST-elevations to be due to recurrent small bowel obstruction. Although intra-abdominal causes of ST-elevation have been reported, our case may be the first to be associated with small bowel obstruction.

## 1. Introduction

Acute ST-elevation myocardial infarction is a medical emergency and is typically associated with high cardiac mortality if brisk intervention is not undertaken. It is important, however, to understand that there are several conditions that may mimic acute ST-elevation myocardial infarction and they should be considered as a differential diagnosis especially in the correct clinical scenario. Our case highlights the importance of understanding the different pathologies, namely, gastrointestinal pathologies, that can be presented as being similar to acute myocardial infarction.

## 2. Case Report

**Figure 1:**
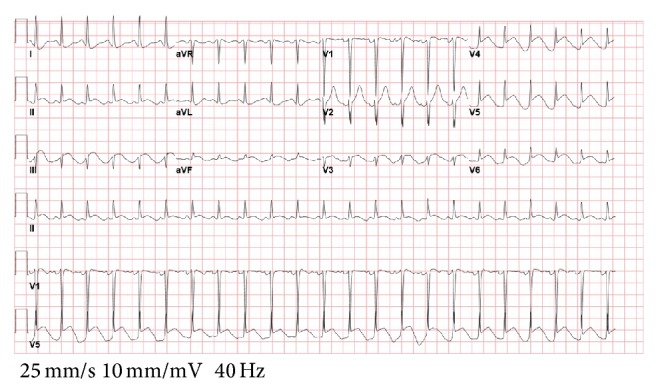


**Figure 2:**
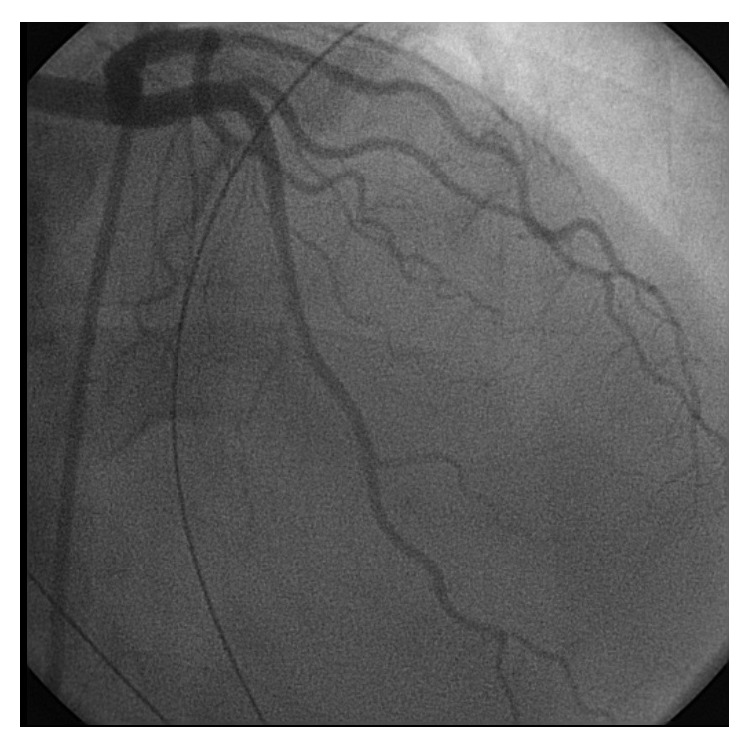


**Figure 3:**
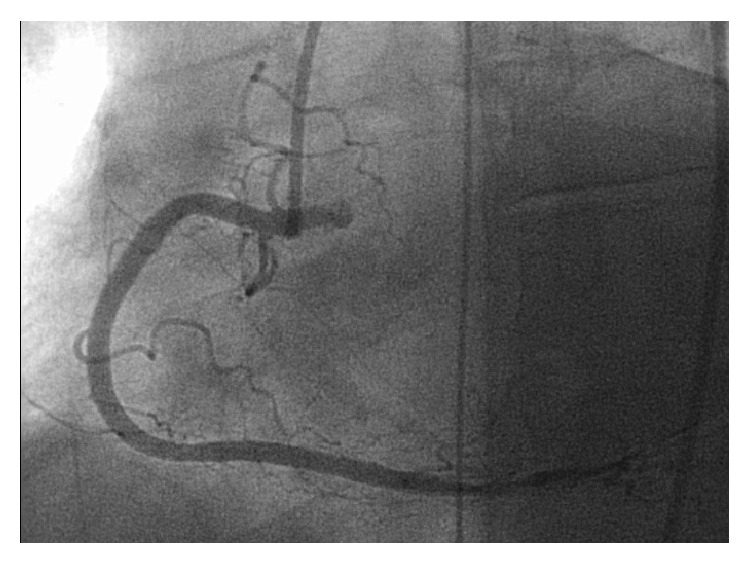


**Figure 4:**
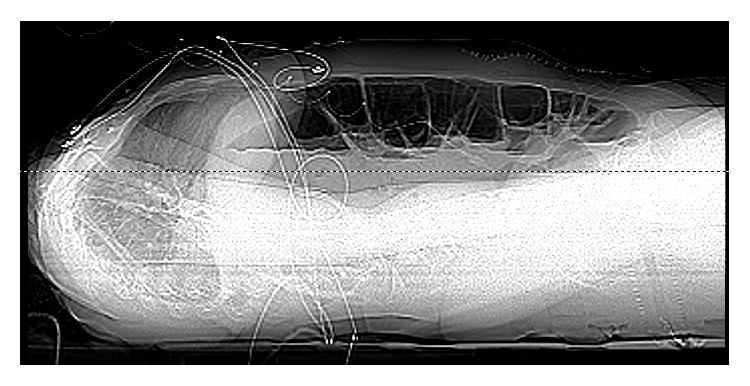


**Figure 5:**
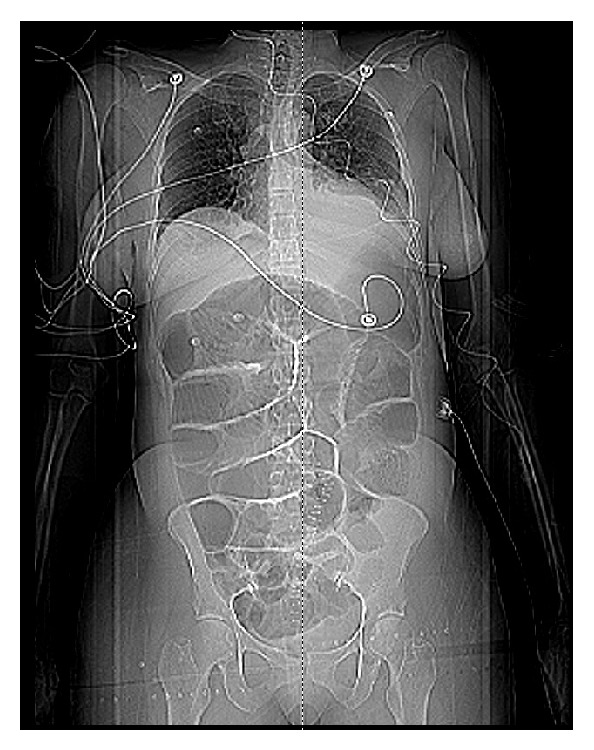


A 42-year-old female with history of endometriosis, status posthysterectomy a number of years prior, presented to our institution complaining of 3-day history of nausea and vomiting. Computed tomography of the abdomen showed bowel obstruction of the distal ileum. The patient was taken for emergent exploratory laparotomy for adhesiolysis and decompression. Postoperatively, during her recovery, the patient started having watery diarrhea and was being monitored on telemetry for persistent sinus tachycardia. Despite intravenous fluids and electrolyte repletion, her condition deteriorated. On day thirteen, ST-elevation MI code was called after an electrocardiogram (Figure 1) suggested the possibility of acute inferolateral myocardial infarction. The patient described epigastric discomfort and shortness of breath but denied chest pain. An immediate bedside Transthoracic Echocardiogram showed predominantly normal left ventricular systolic function; however, inferior wall hypokinesis was noted. Subsequent cardiac catheterization showed normal coronary arteries (Figures 2 and 3) and normal left ventricular contractility by ventriculogram. Serial cardiac enzymes were normal. Alternate causes for the ECG changes were entertained at this point. Thereafter, the patient had a repeat CT scan of the abdomen, which showed severe distention of the stomach and proximal small bowel and recurrent obstruction at the level of distal ileum (Figures 4 and 5). The patient was again taken for emergent exploratory laparotomy and decompression and this time with resection and anastomosis. A total of 2800 cc of fecal material was drained from the small bowel at the time of decompression. Repeat postoperative electrocardiogram (Figure 6) showed normalization of ST segments.

## 3. Discussion

ST-segment elevation myocardial infarction is an emergency during which patient outcomes are directly related to timely intervention and revascularization. However, in cases where patients do not present with typical chest pain, other diagnostic tools such as serial cardiac markers and imaging modalities can help elucidate other causes of ECG findings suggesting acute myocardial infarct. A number of disease presentations may mimic ST-elevation myocardial infarction on ECG [1]. Namely, the most common causes are acute pericarditis, myocarditis, coronary spasm, traumatic brain injury, acute aortic dissection, and ventricular aneurysm.

Few cases of intra-abdominal pathology causing ST-elevation on ECG have been reported. These conditions have been related to pancreas and gallbladder disease [2–4], esophageal pathology [5–9], splenic rupture [10], and hiatal hernia [11]. To date, our case may be the first reported case of small bowel obstruction as a cause for significant ST-elevation on electrocardiogram mimicking acute myocardial infarction. In our case, the patient developed inferolateral ST-segment elevation on a 12-lead ECG which resolved rapidly after surgical decompression of intestinal distension. It is believed that the intra-abdominal distension resulted in the compression of the diaphragmatic surface of the heart and led to the subsequent ECG changes.

## Figures and Tables

**Figure 6:**